# Nutrient Signaling and Lysosome Positioning Crosstalk Through a Multifunctional Protein, Folliculin

**DOI:** 10.3389/fcell.2020.00108

**Published:** 2020-03-03

**Authors:** Natàlia de Martín Garrido, Christopher H. S. Aylett

**Affiliations:** Section for Structural and Synthetic Biology, Department of Infectious Disease, Imperial College London, London, United Kingdom

**Keywords:** folliculin, mTORC1, nutrient signaling, lysosome positioning, Rag GTPases, Rab GTPases

## Abstract

*FLCN* was identified as the gene responsible for *Birt-Hogg-Dubé* (BHD) syndrome, a hereditary syndrome associated with the appearance of familiar renal oncocytomas. Most mutations affecting *FLCN* result in the truncation of the protein, and therefore loss of its associated functions, as typical for a tumor suppressor. *FLCN* encodes the protein *folliculin* (FLCN), which is involved in numerous biological processes; mutations affecting this protein thus lead to different phenotypes depending on the cellular context. FLCN forms complexes with two large interacting proteins, FNIP1 and FNIP2. Structural studies have shown that both FLCN and FNIPs contain longin and *differentially expressed in normal versus neoplastic cells* (DENN) domains, typically involved in the regulation of small GTPases. Accordingly, functional studies show that FLCN regulates both the Rag and the Rab GTPases depending on nutrient availability, which are respectively involved in the mTORC1 pathway and lysosomal positioning. Although recent structural studies shed light on the precise mechanism by which FLCN regulates the Rag GTPases, which in turn regulate mTORC1, how FLCN regulates membrane trafficking through the Rab GTPases or the significance of the intriguing FLCN-FNIP-AMPK complex formation are questions that still remain unanswered. We discuss the recent progress in our understanding of FLCN regulation of both growth signaling and lysosomal positioning, as well as future approaches to establish detailed mechanisms to explain the disparate phenotypes caused by the loss of FLCN function and the development of BHD-associated and other tumors.

## Introduction

In 2018, over 400,000 new cases of kidney cancer were diagnosed worldwide according the Global Cancer Observatory, being the fourteenth most commonly occurring cancer and resulting in more than 175,000 deaths due to a paucity of effective treatments. Kidney cancer is not a unique disease; it encompasses several cancers located in the kidney, each caused by mutations in several different genes, and each requiring specific treatment. The appearance of familiar renal oncocytomas, a specific histological type of kidney cancer, is associated with *Birt-Hogg-Dubé* (BHD) syndrome (Toro et al., 1999), a rare disease with roughly 600 reported families worldwide according to the BHD foundation. BHD syndrome is a hereditary renal cancer syndrome that predisposes individuals to develop cutaneous fibrofolliculomas, lung cysts, spontaneous pneumothorax and, ultimately, kidney tumors (Birt et al., 1977; Toro et al., 1999). The germline mutations found in patients suffering from BHD span the entire *FLCN* gene. *FLCN* has therefore been identified as the gene responsible for BHD (Nickerson et al., 2002). BHD patients carrying a germline mutation in one FLCN allele typically acquire a second hit somatic mutation or loss of heterozygosity in the other wild-type copy over their lifetime. This results in the characteristic BHD renal tumors, in accordance with the two-hit model described for tumor suppressor genes (Knudson, 1971; Schmidt and Linehan, 2018). According to the BHD foundation, approximately 1 in 3 people with BHD develop kidney cancer. To date, most mutations found in *FLCN* in BHD patients result in frameshifts (insertion/deletion), nonsense open reading frames, or the loss of proper mRNA splicing, and are reported in the Leiden Open Variation Data Base (LOVD) (Lim et al., 2010). The predominant result of these mutations is the truncation of the protein, and therefore loss of its associated functions, as typical for a tumor suppressor (Birt et al., 1977; Vocke et al., 2005).

The *FLCN* gene encodes the protein *folliculin* (FLCN) which is 579 amino acids in length and has a mass of 64 kDa in humans. Although no sequence homology has been reported with other known proteins, FLCN is highly conserved across species, being 92% identical to its mouse ortholog, 28% identical to its *D. melanogaster* ortholog and 22% to the *C. elegans* ortholog. Northern blot analysis revealed that, in humans, FLCN is expressed in a wide range of adult tissues, including brain, heart, skin, lung and kidney, as well as fetal lung, liver and kidney (Nickerson et al., 2002). Moreover, homozygous loss of FLCN causes early embryonic lethality, Hasumi et al. (2009) suggesting that FLCN has an important biological role. The identification of FLCN as the tumor suppressor associated with BHD syndrome led several research groups to investigate the mechanism by which the loss of functional FLCN results in kidney cancer. The current consensus is that FLCN is a pleiotropic protein involved in numerous biological processes, including membrane trafficking, energy and nutrient homeostasis, and lysosomal biogenesis (Schmidt and Linehan, 2018). FLCN mutations therefore lead to different phenotypes depending on their cellular context. FLCN forms complexes with two larger *Folliculin interacting proteins* (FNIPs): FNIP1 (Baba et al., 2006) and FNIP2 (Hasumi et al., 2008; Takagi et al., 2008). The same behavior has been found to be recapitulated in yeast, where the respective orthologs, *Lethal with Sec13* (Lst) 7 corresponding to FLCN and Lst4 to the FNIPs (Supplementary Figures 1, 2), also form a complex (Pacitto et al., 2015). Considering the close relationship between the FNIPs and FLCN it is not surprising that they have also been suggested to act as tumor suppressors, as mice deficient in FNIP1 and/or FNIP2 exhibit tumors in several different organs (Hasumi et al., 2015). FNIP1 and FNIP2 were also found to be critical for the tumor-suppressive function of FLCN in kidney tissue, suggesting that the appearance of tumors in BHD patients may be caused by the loss of essential FLCN-FNIP interactions (Hasumi et al., 2015). Additionally, frameshift mutations that would cause premature stop codons in both FNIP1 and FNIP2 have been reported in gastric and colorectal malignancies, supporting a role for FNIP1 and FNIP2 in the development of these cancers (Mo et al., 2019), however further studies are required to clarify the roles of FLCN interacting partners in tumorigenesis. Recent structural studies have shown that the FLCN and FNIP proteins each contain both a longin and a *differentially expressed in normal versus neoplastic cells* (DENN) domain (Nookala et al., 2012; Zhang et al., 2012; Lawrence et al., 2019; Shen et al., 2019), which are protein folds that have been variously implicated in the regulation of small GTPases and membrane trafficking. Accordingly, functional studies support the notion that FLCN-FNIP complex regulates both the Rag and Rab GTPase families (Dodding, 2017; Schmidt and Linehan, 2018), which in turn modulate the key mTORC1 signaling pathway and lysosomal distribution respectively, in a manner dependent on amino acid availability. Here we attempt to summarize our current knowledge of FLCN both in nutrient signaling and in lysosomal positioning.

## The FLCN Complex Regulates the mTORC1 Signaling Pathway Through the Rag Gtpases Based on Nutrient Availability

### The Rag GTPases Communicate the Current Nutrient Availability to mTORC1 Depending on Their Nucleotide Binding State

The *Target of Rapamycin* (TOR) was first identified in *Saccharomyces cerevisiae* cells treated with rapamycin, where it results in irreversible cell cycle arrest (Heitman et al., 1991). Several years later, a *mammalian* TOR (mTOR) homolog (Brown et al., 1994; Sabatini et al., 1994) was identified. mTOR forms the core of two separate multiprotein complexes, mTORC1 and mTORC2, which are differentiated by their complements of accessory proteins (Kim et al., 2002; Loewith et al., 2002; Sarbassov et al., 2004; Laplante and Sabatini, 2012; Shimobayashi and Hall, 2014). Since the discovery of the mTOR complexes, extensive efforts have been made to characterize them and to distinguish their functions. Whereas mTORC2 regulates cell survival, metabolism and cytoskeletal structure (Oh and Jacinto, 2011), mTORC1 functions as a central regulator of metabolism, ensuring that the cell grows only under favorable conditions (Rabanal-Ruiz and Korolchuk, 2018). The dysregulation of the mTORC1 signaling pathway is thereby associated with many forms of cancer and metabolic disorders (Guertin and Sabatini, 2007; Saxton and Sabatini, 2017; Mossmann et al., 2018). The central components of mTORC1 are mTOR, which provides the catalytic core, *regulatory-associated protein of mTOR* (RAPTOR) (Hara et al., 2002; Kim et al., 2002; Loewith et al., 2002), and *mammalian lethal with Sec13 protein 8* (mLST8) (Chen and Kaiser, 2003; Kim et al., 2003), while *proline-rich Akt substrate of 40 KDa* (PRAS40) and *DEP domain containing mTOR interacting protein* (DEPTOR) are minor regulatory components. The central components form a dimeric complex interacting through the N-terminal HEAT repeats of the catalytic core, mTOR (Aylett et al., 2016; Anandapadamanaban et al., 2019). mTOR signaling is dependent on its serine/threonine kinase activity toward target substrates. The mTORC1 complex triggers cell proliferation and cell growth by promoting anabolic processes and suppressing catabolic metabolism through the phosphorylation of key effector proteins such as *ribosomal protein S6 kinase* (S6K) and the translation repressor *eukaryotic translation initiation factor 4E-binding protein* (4E-BP) (Rabanal-Ruiz and Korolchuk, 2018; Kim and Guan, 2019).

**FIGURE 1:**
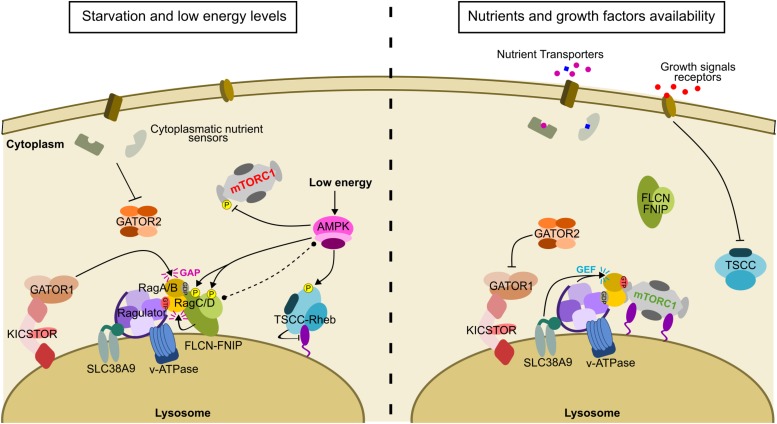
The regulation of the mTORC1 signaling pathway depends on multiple different stimuli. When nutrients are not present mTORC1 is not recruited to lysosomes. Conversely, FLCN is recruited to the lysosome through an interaction with RagA-GDP, downstream of GATOR1 GAP activity (shown in pink). Low energy levels activate AMPK (activation is shown in black arrows) which is thought to phosphorylate Raptor and TSCC, both events having an inhibitory effect on mTORC1 activity (inhibition is shown in black lines with a bar at the end). AMPK is also thought to bind and phosphorylate FLCN-FNIP (putative binding is indicated by a doted black line terminated with black dots). In the reverse situation, when nutrients and growth factors are available, FLCN disperses from the lysosome, after exerting its GAP activity toward RagC, to leave space for mTORC1 recruitment, while GATOR1 is inhibited by GATOR2 and Ragulator and SLC38A9 exert their GEF activity toward RagA. The appropriate Rag GTPase nucleotide state recruits mTORC1 to the lysosome. In parallel, growth factors inhibit TSCC which in turn exposes Rheb on the lysosomal surface to allosterically activate mTORC1.

The level of activation of mTORC1 is dependent on the nucleotide state of three small GTPases: mTORC1 is recruited to the lysosome by heterodimeric *Ras-related GTP-binding protein* (Rag GTPases) (Sancak et al., 2008) where it is catalytically activated by GTP-bound *Ras homolog enriched in brain* (Rheb) GTPase (Long et al., 2005; Sato et al., 2009). Both pathways are necessary, with neither being sufficient to activate mTORC1 independently. It is widely accepted that multiple intracellular and extracellular inputs are integrated to finely regulate the activation of mTORC1 (Laplante and Sabatini, 2012). While external and survival stimuli, such as growth factors or cellular stress, regulate mTORC1 activity through the Rheb axis, signals corresponding to the intracellular nutrient and energy state, such as amino acid availability, are communicated to the Rag GTPases to control the lysosomal localization of mTORC1. However, whether or not some stimuli override others, and whether or not there are diverse activation mechanisms depending on the cell type and context, remains unclear. The main regulator of Rheb is the *TSC complex* (TSCC), consisting of the two large *tuberous sclerosis complex proteins 1* (TSC1) and TSC2, also known as hamartin and tuberin, respectively (Tomasoni and Mondino, 2011), and a smaller protein called TBC1D7 (Dibble et al., 2012). The largest member of the complex, TSC2, acts as a *GTPase activating protein* (GAP) for the small GTPase Rheb, promoting its inactive GDP-bound conformation, and therefore as a negative regulator of mTORC1 (Tee et al., 2002; Zhang et al., 2003). Nutrients, in particular amino acids, activate lysosome-associated signaling machinery to recruit mTORC1 through the Rag GTPases, which directly interact with mTORC1 in an amino acid dependent manner (Kim et al., 2008; Sancak et al., 2008). The regulation of the Rag GTPases is highly sophisticated, involving several multiprotein complexes acting as GAPs and *guanine nucleotide exchange factors* (GEFs): *GAP Activity Toward Rags* (GATOR) 1, a GAP for RagA/B, FLCN-FNIP, a GAP for RagC/D, GATOR2, an inhibitor of GATOR1, *KPTN, IKTFG2, C12orf66 and SZT2* (KICSTOR), which is thought to regulate the GATOR1 complex, and the pentameric *Rag and mTORC1 regulator* (Ragulator) complex, which acts as a GEF for RagA/B (Ramlaul and Aylett, 2018). Overall, mTORC1 signaling represents a sophisticated pathway through which several signals converge to regulate most major cellular functions involved in cell growth (Figure 1). Therefore, is not unexpected that many years after its discovery, our understanding of the mechanism of action and regulation of mTOR is constantly evolving.

While it is widely accepted that mTORC1 activation depends on nutrient availability (Blommaart et al., 1995; Hara et al., 1998), how these stimuli trigger mTORC1 signaling is less clear. The latest evidence suggests that amino acids are sensed through different mechanisms from inside and outside the lysosome via certain human proteins known to act as metabolic sensors. These include cytoplasmic nutrient sensors (Sestrins, CASTORs and SAMTOR), amino acid transporters (SLC38A9) and the v-ATPase within the lysosomal membrane (Rabanal-Ruiz and Korolchuk, 2018; Ramlaul and Aylett, 2018). All these signals converge upon the Rag GTPases, which are anchored to the lysosome via the Ragulator complex (Sancak et al., 2010; Bar-Peled et al., 2012), and which together make up the essential platform for the recruitment of mTORC1 to the lysosome.

In mammals, there are four Rag GTPases that form heterodimers between two sub-types (Sekiguchi et al., 2001). RagA and RagB were firstly discovered as members of the Ras superfamily of small GTPases and homologs of yeast Gtr1 (Schürmann et al., 1995). RagC and RagD were identified as Gtr2 orthologs, completing the Rag family (Sekiguchi et al., 2001). Human RagA is paralogous to RagB, with 90% sequence identity, while human RagC is paralogous to RagD, with 81% sequence identity (Nicastro et al., 2017). Rag family members possess sequence elements that are common to all GTPases of the Ras family, including the P-loop and *switch regions I* (SWI) and *II* (SWII), and guanine binding motifs with some important differences (Supplementary Figure 3A). The switch regions adopt open or closed conformations upon GTP or GDP binding (Wittinghofer and Vetter, 2011; Jeong et al., 2012) in order to transmit to mTORC1 the nutrient status of the cell. Their guanine binding motifs, G2 and G3, are also divergent, with an asparagine substituted for a histidine in G2, and an isoleucine instead of an alanine in G3 (Schürmann et al., 1995; Sekiguchi et al., 2001; Sekiguchi et al., 2014). Another remarkable feature of the Rag GTPases is their large *C-terminal domains* (CTDs), which are responsible for the assembly of functional heterodimers containing RagA or B combined with RagC or D (Sekiguchi et al., 2001; Gong et al., 2011). The Rag heterodimer is pseudo-two-fold symmetric, with an overall U-shape (Supplementary Figure 3B). Heterodimerization is achieved by direct contacts between the RagA/B and RagC/D CTDs, whereas no direct interaction is established between their *nucleotide binding domains* (NBDs), a novel architecture previously unreported for any GTPase (Gong et al., 2011; Jeong et al., 2012). The NBDs are located on the same side of the complex but face in opposite directions.

Amino acid availability promotes the active Rag GTPase nucleotide configuration, RagA/B-GTP and RagC/D-GDP, which is capable of recruiting mTORC1 through a direct interaction with Raptor (Sancak et al., 2008). Conversely, when amino acid levels are low, the opposite Rag GTPase nucleotide binding state, in which RagA/B is GDP-bound and RagC/D is GTP-bound, is favored, which results in the release of mTORC1 from the lysosome. The recent determination of the structures of two mTORC1-Rag complexes has shed light on the molecular basis of Rag GTPase signal transduction. The first study determined the Raptor-Rag-Ragulator architecture, revealing that Raptor first binds to RagA-GTP in a transient interaction, and can only attach fully to the Rag GTPase heterodimer when RagC is GDP-bound (Rogala et al., 2019). Concurrently, the structure of the mTORC1-RagA-RagC complex demonstrated that the α-solenoid domain of Raptor contacts the Rag GTPase NBDs, leaving the CTDs with a limited interaction surface with Raptor (Anandapadamanaban et al., 2019). The RagA-GTP switch and inter-switch regions are more ordered than RagC-GDP because they form more extensive contacts with mTORC1 (Supplementary Figure 3C). In the reverse situation (GDP-RagA-RagC-GTP), the Rag GTPase complex would not bind to Raptor, because the switch and inter-switch regions of RagA-GDP would be disordered (Supplementary Figure 3D) and RagC-GTP residues analogous to RagA Raptor-binding regions are not conserved (Anandapadamanaban et al., 2019). Structural analysis of the mTORC1-RagA-RagC complex also revealed that, while the Rag GTPases undergo multiple conformational changes upon Raptor binding, the binding of the Rag heterodimer does not cause any conformational changes within mTORC1. This supports the current hypothesis that mTORC1 is recruited by the Rag-Ragulator platform to lysosomes, where it is allosterically activated by Rheb-GTP. Combination of the new mTORC1-Rag GTPase structure with previously published structures of mTORC1-Rheb (Yang et al., 2017) and Ragulator bound to RagA-RagC (de Araujo et al., 2017; Yonehara et al., 2017) provides sufficient information to build a structural model of the active mTORC1 super-complex at the lysosome. This model would allow the simultaneous interaction of mTORC1 with both the Rag GTPases and Rheb, while leaving the catalytic site facing the cytosol to allow mTORC1 to fulfill its function as a kinase and phosphorylate downstream targets (Anandapadamanaban et al., 2019).

The double nucleotide-bound activating conformation, with both GTP and GDP bound on the Rag heterodimer, especially the requirement for GDP state in one active site and the linked architecture, are features unique to the Rags among GTPases (Mishra and Lambright, 2016). Kinetic assays with radiolabeled nucleotides have implied that this unique architecture allows crosstalk between the two subunits: when one GTPase domain is bound to GTP, the heterodimer is in a locked conformation that suppresses the association of a second GTP in the opposing domain or induces fast hydrolysis if this exchange occurs (Shen et al., 2017). This inter-subunit communication provides a singular mode of regulation of GTPases which provides more intermediate states that participate in the precise regulation of mTORC1 signaling pathway. Cryo-EM structural data combined with *hydrogen/deuterium exchange mass spectrometry* (HDX-MS) of mTORC1 in complex with the Rag GTPase heterodimer revealed the means by which the nucleotide state of the NBD is sensed by the corresponding CTD (Anandapadamanaban et al., 2019), which undergoes conformational changes depending on the Rag GTPase nucleotide state, providing the structural basis for the Rag inter-subunit crosstalk. Moreover, the heterodimeric setting furnishes the Rag GTPases with additional conformational possibilities in comparison to the situation for monomeric small GTPases, to satisfy their requirements for interactions with multiple regulators. The basal GTPase activity of the Rag GTPase heterodimer is lower than that of other Ras-family members (Frech et al., 1994; Shen et al., 2017), therefore they are more dependent on their GAPs in order to switch between their active and inactive settings in response to nutrient availability. There are two protein complexes with GAP activity toward the Rag GTPases: GATOR1 for RagA/B (Bar-Peled et al., 2013; Panchaud et al., 2013) and FLCN-FNIP for RagC/D (Petit et al., 2013; Tsun et al., 2013).

To exert their amino-acid dependent mTORC1-recruiting function, the Rag GTPases are localized to the lysosome, interacting with the lysosomal membrane protein *Late Endosomal/Lysosomal Adaptor, MAPK and mTOR activator/regulator* (LAMTOR) 1 (Sancak et al., 2010), a part of the “Ragulator” complex (Bar-Peled et al., 2012). The Ragulator complex is formed by five subunits; LAMTOR1 (p18), LAMTOR2 (p14), LAMTOR3 (MP1), LAMTOR4 (HBXIP) and LAMTOR5 (C7orf59). This complex acts as a scaffold for the Rag GTPases, with LAMTOR1 being the key component required to attach them to the lysosome via myristoylation and palmitoylation sites within its N-terminus (Nada et al., 2009; Sancak et al., 2010; Bar-Peled et al., 2012). LAMTOR 2, 3, 4, and 5 contain roadblock domains that form heterodimers, LAMTOR2 with LAMTOR3 and LAMTOR4 with LAMTOR5. The assembled Ragulator complex has an elongated shape with LAMTOR1 α-helices encircling LAMTOR 2-3 and LAMTOR 4-5 heterodimers (Mu et al., 2017). Ragulator is not merely a platform for the proper localization of the Rag GTPases to the lysosome, but also possesses GEF activity to promote their exchange to the active nucleotide state. Ragulator was firstly identified as a GEF for Rag A/B as it accelerates the release of both GDP and GTP and favors the active Rag GTPase nucleotide state (Bar-Peled et al., 2012). Additionally, recent kinetic studies have found that SLC38A9, a lysosomal amino acid sensor, cooperates with Ragulator through an atypical GEF mechanism (Shen and Sabatini, 2018). The structure of the full complex with the Rag GTPases supports the function of Ragulator; the C-terminal Roadblock domain of RagC binds to the LAMTOR2-3 heterodimer (de Araujo et al., 2017; Su et al., 2017; Yonehara et al., 2017), thereby exposing the NBDs as required for their interaction with Raptor. Interestingly, the Roadblock domains found in LAMTOR subunits 2–5 are very common in GTPase interacting partners (Levine et al., 2013). Ragulator thereby functions both as a platform for, and a component of, the regulatory elements of the nutrient signaling pathway, which includes the FLCN-FNIP complex.

### The FLCN Complex Acts as a GTPase Activating Protein to Finely Modulate Rag GTPase Nucleotide Binding and Transmit the Nutrient Status to mTORC1

Together with the GATOR complexes, the FLCN complex acts as a GAP for the Rag GTPases (Figure 1). FLCN-FNIP controls the nucleotide state of RagC/D, promoting the RagC/D-GDP state, and therefore acting as a positive modulator of mTORC1. Under starvation conditions, FLCN-FNIP is recruited to lysosomes where it interacts with RagA-GDP, whereas when nutrient levels are recovered, the complex dissociates and returns to the cytosol (Petit et al., 2013; Tsun et al., 2013), allowing GTP-bound RagA/B to recruit mTORC1 to the lysosome where it can be fully activated by GTP-Rheb (Figure 1). RagA and RagC coimmunoprecipitated with FLCN that had been co-expressed with FNIP2, suggesting that a FLCN-FNIP complex is required for either of the two proteins to interact with the Rag GTPases (Petit et al., 2013; Tsun et al., 2013). This behavior is recapitulated in yeast, where the respective orthologs, Lst7 and Lst4, localize to the vacuolar membranes when cells are starved, but rapidly dissociate from this compartment when amino acid levels are restored (Pacitto et al., 2015; Péli-Gulli et al., 2015). Loss of either Lst4 or Lst7 similarly decreases TORC1 activity in yeast cells, suggesting that they share a common biological activity toward TORC1 (Péli-Gulli et al., 2015).

The identification of FLCN-FNIP as a critical component of the lysosome-localized machinery for the proper transduction of signals of amino acid availability to mTORC1 through the Rag GTPases, spurred efforts to understand the functional relevance of the FLCN-FNIP recruitment to this compartment. FLCN-FNIP complex possesses GAP activity toward RagC/D, a fact established through GTP hydrolysis assays using different combination of Rag mutants in HEK293T cells, and hence promotes RagC/D-GDP occupancy, activating mTORC1 in an amino acid dependent manner (Tsun et al., 2013). A FLCN-FNIP2 complex is required to exert GAP activity toward RagC/D, as the individual proteins are insufficient to catalyze nucleotide exchange. This is borne out by the presence of longin domains on both FLCN and FNIP proteins, similar to those found in GATOR1 (Shen et al., 2018) which acts as a GAP for RagA/B. Indeed, a recent study proposed that FLCN-FNIP GAP activity occurs downstream of GATOR1 (Meng and Ferguson, 2018); when amino acid levels are low, the GAP activity of GATOR1 promotes the GDP-RagA/B state, and FLCN/FNIP is then recruited to the lysosome to act as a GAP toward RagC/D. Although the FLCN-FNIP GAP activity toward RagC/D identifies this complex as a positive regulator of mTORC1, renal tumors from BHD patients containing FLCN germline mutations showed increased mTORC1 activity (Baba et al., 2008; Hasumi et al., 2009), as well as higher levels of phosphorylation of mTORC1 substrates (Khabibullin et al., 2014). In light of these counterexamples, it has been suggested that the role of FLCN loss of function in renal tumorigenesis may be more sophisticated, not only involving mTORC1 activation, but also other signaling pathways that in turn can affect mTORC1 signaling.

### A Potential Interaction of FLCN-FNIP With AMPK

**FIGURE 2:**
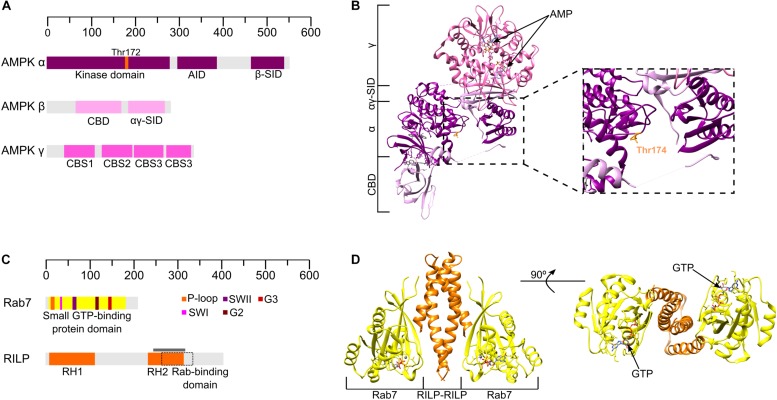
Structures of AMPK and the Rab7-RILP complex. **(A)** Schematic domain organization of human AMPK subunits based on the structure represented in panel B (α1 β1 γ1). AID, autoinhibitory domain. SID, C-terminal subunit interacting domain. CBM, beta sheet rich carbohydrate binding module. CBS, cystathionine b synthase. **(B)** Crystal structure of human AMPK (PDB: 6C9F) at 2.65 Å resolution co-crystallized in the presence of R734, staurosporine, and AMP. The panel *inset* on the right shows how Thr174 is protected from dephosphorylation. **(C)** Domain organization of human Rab7 and RILP. Colored boxes on Rab7 indicate typical elements for GTPases, according to the legend. The gray line indicates the part of RILP that is shown in the crystal structure and the doted square box represents the Rab-binding domain of RILP. **(D)** Crystal structure of Rab7-RILP human proteins (PDB: 1YHN) from two different views.

While we have discussed mTORC1 activation with respect to nutrient availability extensively, this kinase also responds to other stimuli, including energy availability. Low energy conditions are normally characterized by high AMP:ATP ratios, which allosterically activates another central metabolic regulator, the *AMP-activated protein kinase* (AMPK) (Carling, 2004; Shackelford and Shaw, 2009). AMPK is a heterotrimeric kinase formed by a catalytic core, the α subunit, and two regulatory subunits; β and γ (Figures 2A,B). There are multiple gene products for each subunit (α1, α2; β1, β2; γ1, γ2, γ3) which can combine to form twelve different heterotrimers (Stapleton et al., 1994; Thornton et al., 1998; Cheung et al., 2000). The α subunit contains a typical serine/threonine protein kinase catalytic domain (Hanks et al., 1988) as well as several phosphorylation sites, with α1/α2-Thr172/174 being essential for AMPK activity (Stein et al., 2000) (Figure 2A). Multiple crystal structures of the complete kinase complex support a functional role for Thr172/174: binding of AMP or other synthetic activators protects this threonine residue from dephosphorylation and, therefore, AMPK inactivation through phosphatase activity (Figure 2B, *inset*) (Xiao et al., 2011, 2013; Calabrese et al., 2014; Yan et al., 2019). Two upstream kinases, *Liver kinase B1* (LKB1) and *calmodulin kinase kinase* (CAMKK), are responsible for the activation of AMPK by phosphorylation of Thr172/174, depending on the cellular context (Woods et al., 2003; Shaw et al., 2004). Activated AMPK increases ATP production through promotion of catabolic pathways and the inhibition of synthetic pathways that consume ATP, in a manner antagonistic to mTORC1 activity. Additionally, AMPK inhibits mTORC1 both directly, through the phosphorylation of its component Raptor (Gwinn et al., 2008), and indirectly, by phosphorylation and activation of TSC2 (Inoki et al., 2002). Crosstalk between mTORC1 and AMPK provides a more specific mechanism by which cell growth may be coordinated according to environmental conditions.

However, inhibition of mTORC1 by AMPK is not the only relationship between these two signaling pathways. Coimmunoprecipitation experiments have demonstrated that FNIP1 can interact with all subunits of AMPK, *in vitro*, and that FLCN is not essential for FNIP-AMPK binding (Baba et al., 2006). FNIP2 was also identified as a FLCN interacting partner that binds AMPK (Hasumi et al., 2008; Takagi et al., 2008). Although it is widely accepted that FLCN binds to AMPK through the FNIPs, the functional relevance of this interaction remains unexplained. It is notable that FNIPs can be phosphorylated by AMPK and both FLCN and FNIP phosphorylation levels are affected by both AMPK signaling and amino acid starvation, which in turn inhibit mTORC1 (Baba et al., 2006; Takagi et al., 2008). Interestingly, FLCN phosphorylation appears to affect complex formation; FNIP1 and FNIP2 preferentially binding phosphorylated FLCN (Baba et al., 2006; Takagi et al., 2008). Additionally, the interaction between the FLCN complex and AMPK has been linked to the induction of apoptosis (Lim et al., 2012) and increased *FLCN* expression is associated with AMPK-dependent dephosphorylation of *Transcription Factor EB* (TFEB), which causes its translocation to the nucleus independently of mTORC1 (Collodet et al., 2019), both examples connecting FLCN-FNIP-AMPK with the coordination of metabolism. Finally, both FLCN and FNIP1 have been suggested as negative regulators of AMPK as depletion of FLCN has been found to constitutively activate AMPK (Possik et al., 2014, 2015; Yan et al., 2014; El-Houjeiri et al., 2019) and mutations affecting FNIP1 are associated with higher AMPK activity (Siggs et al., 2016), suggesting that FLCN and FNIP may cooperate to modulate AMPK. In the same way, phosphorylated FNIP1 can bind to chaperone Hsp90, which indeed, regulates proper folding of AMPK subunits and some mTORC1 pathway components, such as Raptor or mTOR itself, suggesting another function for FNIP1 in the regulation of AMPK and mTORC1 pathways (Woodford et al., 2016; Sager et al., 2018, 2019). All this evidence strongly suggests a role for FLCN-FNIP in coordinating cellular metabolism through its effects on both the mTORC1 and AMPK signaling pathways, however, further studies are eagerly awaited to clarify the functional relevance of the FLCN-FNIP-AMPK interaction.

## How Nutrients Coordinate the Distribution of Lysosomes Within the Cell

### The Lysosome Is a Key Organelle for Activation of the mTORC1 Signaling Pathway

The consensus view of mTORC1 signaling is that it is only active when both the Rag GTPase and the Rheb GTPase axes are fully activated, meaning that mTORC1 needs to be recruited to lysosomes to be fully operational. However, within the signaling pathway this organelle is not solely a platform for the proper assembly of mTORC1 regulatory elements. It is much more deeply involved in the shift between anabolism and catabolism, a function that has presumably evolved as a result of its pivotal role in autophagy. The relationship between mTORC1 and the lysosome is crucial for the control of lysosomal function and provides this organelle with the capacity to sense nutrient availability and generate a cellular response (Settembre and Ballabio, 2014; Rabanal-Ruiz and Korolchuk, 2018). Lysosomes comprise a single-lipid bilayer membrane containing a set of luminal hydrolases responsible for the degradation of a wide range of substrates, including sugars, lipids proteins and nucleic acids (de Duve et al., 1955; de Duve, 2005; Sleat et al., 2013). Lysosomal hydrolases have an acidic optimum pH, therefore the lysosomal lumen needs to be acidic for the proper function of this organelle, promoting protein unfolding (Xu and Ren, 2015). The acidic pH of the lysosomal lumen is maintained by the v-ATPase, introduced earlier as a putative amino acid sensor, a large channel that pumps protons across the lysosomal membrane toward the lumen (Mindell, 2012). Intracellular and extracellular substrates can both be degraded by lysosomes. While extracellular macromolecules reach the lysosome through endocytosis (Conner and Schmid, 2003), cytoplasmic macromolecules or damaged proteins are processed through the autophagic pathway (He and Klionsky, 2009; Kaur and Debnath, 2015).

The function of lysosomes is dependent on their ability to move bidirectionally between the centrosomal and peripheral areas of the cell along linear tracks formed by microtubules (Matteoni and Kreis, 1987). The balance between centrosomal and peripheral lysosomal transport defines the dynamic cytoplasmic distribution of lysosomes, which are normally well-distributed over the cytoplasm with a small degree of enrichment around the nucleus, close to the *microtubule organizing center* (MTOC) and the Golgi apparatus (Korolchuk et al., 2011). There are two different well-established pathways that control lysosome transport toward the centrosomal and the peripheral region of the cell respectively. Transport toward the plus end of microtubules, usually located at the cell periphery, is regulated by kinesins. The *BLOC-one-related complex* (BORC) mediates this process by recruiting the small GTPase *Arf-like8b* (Arl8b), which in turn binds to the adaptor protein *SiFA and kinesin interacting protein* (SKIP) to finally recruit kinesin-1, the motor responsible for transport (Rosa-Ferreira and Munro, 2011; Pernigo et al., 2013). In parallel, Rab7 and *FYVE and coiled-coil domain containing 1* (FYCO1) can also recruit kinesin-1 to promote plus end movement of lysosomes (Pankiv et al., 2010; Raiborg et al., 2015). Conversely, transport toward the minus end of microtubules, which are located within the perinuclear or centrosomal region of the cell, is regulated by dynein-mediated pathways. Rab7 initiates this process by binding to *Rab interacting lysosomal protein* (RILP), which ultimately recruits cytoplasmic dynein (Cantalupo et al., 2001; Jordens et al., 2001). Alternatively, other studies have shown that the Golgi-located Rab34 and Rab36 GTPases can also recruit RILP, hence affecting lysosome distribution by promoting perinuclear clustering (Wang and Hong, 2002; Goldenberg et al., 2007; Chen et al., 2010).

It is noticeable that the intracellular distribution of lysosomes correlates with nutrient availability: in HeLa cells, starvation promotes not only the inhibition of mTORC1 activity but also results in a lysosomal clustering on the perinuclear region (Korolchuk et al., 2011). Additionally, withdrawal of nutrients promotes the translocation of the MiT family transcription factors TFEB and *Transcription Factor Binding To IGHM Enhancer 3* (TFE3) to the nucleus to induce lysosomal biogenesis and autophagosome formation (Sardiello et al., 2009; Settembre et al., 2011). During autophagy, vesicles containing damaged protein and cell debris are transported to the minus end of microtubules, and therefore the perinuclear region of the cell, where they are finally fused to the lysosomes responsible for the degradation of their contents. The promotion of autophagy links the inhibition of mTORC1 activity during starvation to activation of catabolic processes through lysosomal activity (Rabanal-Ruiz and Korolchuk, 2018; Kim and Guan, 2019). Given the availability of amino acids and nutrients the situation is reversed, the minus end transport of lysosomes being inhibited with the result that instead lysosomes are located close to the plasma membrane (Korolchuk et al., 2011). Amino acid availability activates mTORC1, which in turn phosphorylates TFEB and TFE3, preventing their translocation to the nucleus, and hence inhibiting lysosomal biogenesis (Martina et al., 2012; Roczniak-ferguson et al., 2012; Settembre et al., 2012). Additionally, amino acids promote the recruitment of kinesin-1 adaptor *FYVE And Coiled-Coil Domain Containing 1* (FYCO1) to lysosomes through interaction with PtdIns3 (Nobukuni et al., 2005; Hong et al., 2017), and the formation of contact sites between lysosomes and the *endoplasmic reticulum* (ER) through Rab7-Protrudin-PtdIns3 binding. Within these lysosomes-ER contact sites, the transfer from Protrudin to FYCO1 allows lysosomes to be loaded onto kinesin-1, therefore promoting transport toward to cell periphery (Raiborg et al., 2015). During starvation, it is clear that lysosomes are clustered toward the centrosomal region of the cell to favor catabolic processes such as autophagy, however, the evolutionary reason for their peripheral location upon nutrient addition remains ambiguous. It has been suggested that lysosomes cluster on the plasma membrane to be closer to the site of growth factor signaling for the full activation of mTORC1 (Korolchuk et al., 2011) and that growth factors are insufficient to activate mTORC1 unless lysosomes are close to the plasma membrane (Hong et al., 2017). Supporting such a relationship between lysosome positioning and mTORC1 activation, immunoblotting assays in HEK293 cells demonstrated that depletion of microtubule motors appeared to disrupt Rag GTPase-dependent mTORC1 activation (Li and Guan, 2013). Overall, there is clear evidence that the lysosomal positioning and mTORC1 signaling pathways are intimately linked, however further studies are needed to shed light on the specific mechanisms by which this is achieved.

### The FLCN Complex Acts as the Intersection Between Nutrient Signaling and Lysosome Positioning

We have covered the means by which the FLCN-FNIP complex is recruited to the lysosome upon starvation, where it interacts with the Rag GTPases and has GAP activity toward RagC (Petit et al., 2013; Tsun et al., 2013), thus playing a crucial role in the proper recruitment of mTORC1 to the lysosome. However, the interaction with the Rag GTPases to recruit mTORC1 is not the only lysosome-related function of FLCN. The loss of FLCN inhibits the phosphorylation of transcription factors TFEB and TFE3, promoting their nuclear translocation and activating lysosome biogenesis (Hong et al., 2010; Martina et al., 2014). Supporting this notion, structure-function analysis have suggested that the role of FLCN in regulating TFEB and TFE3 localization is dependent on the GAP activity of FLCN toward RagC (Lawrence et al., 2019), whereas immunoblotting assays in mammalian and nematode cells suggested that the loss of FLCN drives TFEB and TFE3 nuclear localization independently from the canonical mTORC1 pathway (El-Houjeiri et al., 2019). On the other hand, FLCN-deficient human cells appear to have impaired autophagosome maturation (Dunlop et al., 2014), suggesting a role for FLCN in autophagy regulation, which has also been reported during studies of the AMPK-FLCN functional relationship (see Section “A Potential Interaction of FLCN-FNIP With AMPK”).

Additionally, immunofluorescence analyses of HeLa cells demonstrated that upon starvation, FLCN is not only recruited to the lysosomes but also that perinuclear clustering of this organelle occurs. Pull-down assays have shown that FLCN can interact with both Rab7 and Rab34, which share RILP as an interaction partner (Starling et al., 2016), suggesting that the formation of a FLCN-RILP-Rab34 complex could be the reason for the perinuclear clustering of lysosomes and identifying a functional connection between lysosomal positioning and the mTORC1 pathway. The formation of a FLCN-Rab34/7-RILP complex is supported by the fact that both FLCN and FNIP have longin and DENN domains, known to be commonly found in GEFs for the Rab GTPases (Levivier et al., 2001). FLCN seems to directly interact with RILP through its C-terminal DENN domain (Starling et al., 2016), a different region from that responsible for its interaction with the Rag GTPases, suggesting a potential role for FLCN-DENN domain in the regulation of lysosomal distribution. The interaction between Rab7 and RILP has previously been studied and a crystal structure is available (Wu et al., 2005), showing that GTP-bound Rab7 binds to RILP through its effector-interacting switch regions, usually recognized by most Rab effectors (Wu et al., 2005) (Figures 2C,D). No GEF or GAP activity has been reported for FLCN-FNIP toward Rab34, although it has been reported that FLCN regulates Rab7 GTPase, acting as a GAP to mediate lysosome-mediated degradation of EGFR (Laviolette et al., 2017). Currently, the functional relevance of the FLCN-RILP-Rab interaction appears to be the promotion of Rab-RILP assembly to promote clustering of lysosomes around the nucleus during nutrient insufficiency, however, structure-function studies are awaited to reveal further details about this significant interaction. Interestingly, there are also reported interactions between FLCN and other Rab GTPases, a family of proteins usually involved in intracellular membrane trafficking (Hutagalung and Novick, 2011). *In vitro* guanine nucleotide exchange assays have suggested that FLCN possesses GEF activity toward Rab35 (Nookala et al., 2012) and that it can bind to this small GTPase through its C-terminus to Rab35, to regulate EGFR intracellular trafficking (Zheng et al., 2017). Although cell-based assays have been unable to validate FLCN GEF activity toward Rab35, its interaction with Rab35 in EGFR regulation provides another example of FLCN linking membrane trafficking to cell growth. Additionally, FLCN was reported to bind to Rab11 via its C-terminal DENN domain and can also promote the Rab11-PAT1 interaction (Zhao et al., 2018). Although no GEF activity of FLCN toward Rab11 has been identified, the FLCN-Rab11 interaction suggests that, apart from tethering the Rags for a proper recruitment of mTORC1 in the lysosome, FLCN may have other roles in the maintenance of the amino acid signal level within the lysosome through amino acid transporters, such as PAT1.

**FIGURE 3:**
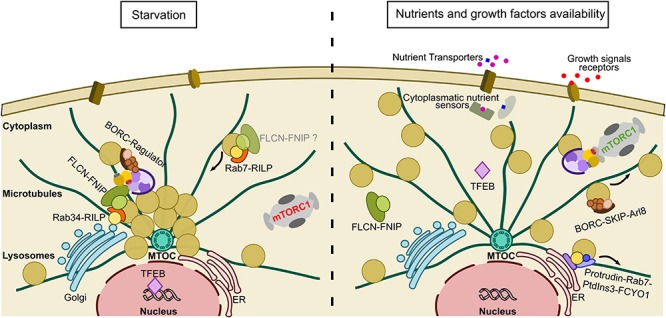
Lysosomal repositioning in response to nutrient availability. Under starvation conditions, mTORC1 activity is abolished and lysosomes cluster in the perinuclear region to favor catabolic processes such as autophagy. Lysosomal clustering around the nucleus is favored by FLCN-RILP-Rab34 contacts on the Golgi and the inhibitory BORC-Ragulator interaction. At the same time, TFEB is relocalized to the nucleus to favor lysosomal biogenesis. When nutrient levels recover, FLCN disperses from the lysosome and the BORC-Ragulator interaction is broken. Kinesin dependent pathways, such as PtdIns3-Rab7-Kinesin and BORC-Arl8-Kinesin, are promoted to favor dispersal of the lysosomes. In parallel, TFEB relocalizes to the cytoplasm, where it is non-functional. Nutrients promote the recruitment of mTORC1 to the lysosome through the Rag GTPases, which now occurs closer to the plasma membrane allowing full activation by growth factors through the Rheb axis.

Interestingly, FLCN is not the only mTORC1 component related to the lysosomal machinery. The Ragulator complex, an essential multiprotein complex for mTORC1 recruitment to the lysosome, has been reported to interact with BORC to inhibit lysosomal transport toward the cell periphery upon starvation (Filipek et al., 2017; Pu et al., 2017). Furthermore, RILP was shown to directly interact with the v-ATPase and regulate its activity (De Luca et al., 2014), and mTORC1, itself, has been suggested to regulate v-ATPase, both examples showing potential mechanisms coupling lysosomal positioning and nutrient signaling (Peña-Llopis et al., 2011). Overall, lysosomal positioning and mTORC1 signaling are clearly interconnected with the nutrient signaling response. Moreover, several studies have reported that they share regulatory components (Figure 3). Accordingly, multiple FLCN-Rab relationships have been reported that directly link the mTORC1 pathway to the lysosomal positioning components, providing a mechanistic explanation for the intimate relationship between this organelle and nutrient signaling. Therefore, structure-function studies of the full FLCN-RILP-Rab complex are urgently needed to fully understand the role of FLCN in the intersection of mTORC1 pathway and lysosome positioning.

## The Structural Organization of the FLCN-FNIP Complex and its Relationship to its Function

Since the discovery of FLCN as the causative gene for BHD syndrome, subsequent work has established that FLCN protein can form complexes with FNIP1 (Baba et al., 2006) and FNIP2 (Hasumi et al., 2008; Takagi et al., 2008), with the same behavior confirmed for their respective orthologs in yeast, Lst7 and Lst4 (Pacitto et al., 2015). Both FNIPs display substantial sequence identity, with 74% sequence similarity between human orthologs, and are conserved across species, from human to *C. elegans*, with the exception of the last C-terminal region which remains more variable. The two isoforms exhibit similar expression patterns, with some specific differential expression of FNIP2 in fat, liver and pancreatic tissue types, which may imply a specific function for FNIP2 in metabolic tissues, potentially involving the non-conserved C-terminal regions (Hasumi et al., 2008). FNIP1 and FNIP2 have also been reported to form multimeric complexes between and among one another (Hasumi et al., 2008) and both FLCN and AMPK were detected in all co-immunoprecipitants containing these multimers. Interestingly, homo- and hetero- FNIP1/2-multimers also exhibited an ability to form FNIP1/FNIP2/FLCN/AMPK complexes, however, further investigation is required to clarify the implication of these complexes for the function of FLCN.

**FIGURE 4:**
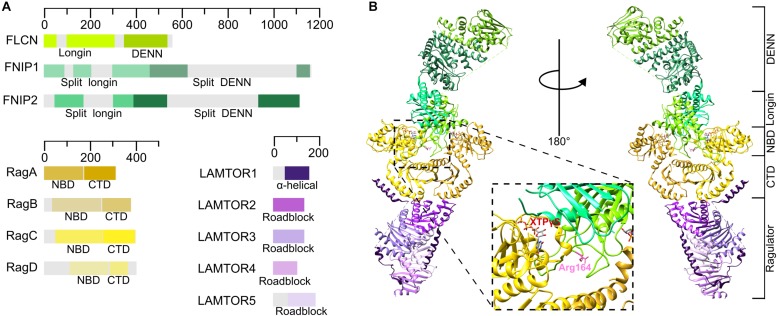
The organization of the FLCN-FNIP complex at the lysosomal membrane. **(A)** Schematic diagram of human FLCN, FNIP, Rag GTPases and Ragulator proteins. **(B)** Full Structure of the FLCN-FNIP-Rag GTPases-Ragulator complex (PDB: 6NZD) from two different views. The *inset* shows that Arg164 of FLCN and the nucleotide bound to RagC are pointing different directions, thus making less plausible that this is the conformation of the complex with GAP activity.

Until recently, no structure of any complete FLCN-FNIP complex was available. After many failed attempts to crystallize the full length protein, the first insights into the FLCN structure were provided by the determination of the crystal structure of the FLCN C-terminal (amino acids 341–566) region to 2 Å resolution (Nookala et al., 2012). This domain has an αβ architecture sharing structural similarity with the DENN family proteins. Comparative studies with the DENN1B full DENN module predicted a longin-like domain at the N-terminal end of FLCN (Figure 4A). The FNIP proteins are much larger (in humans, 130 kDa for FNIP1, 122 kDa for FNIP2) and first sequence and structural studies implied that they also contained DENN domains (Zhang et al., 2012). In contrast to FLCN, both the N-terminal longin and the C-terminal DENN domains encode larger, apparently unstructured, regions within them (Figure 4A) which contain putative phosphorylation sites possessing large numbers of serine and threonine residues, suggesting a post-translational regulatory role for these unstructured regions. The crystal structure of the N-terminal FNIP yeast ortholog, Lst4 (PDB: 4ZY8) (Pacitto et al., 2015), revealed that this domain has a classical longin architecture formed by a core of five β-sheets with a single short α-helix on one face and two longer α-helices on the other, thus confirming the DENN family membership of Lst4 and the FNIP proteins (Supplementary Figure 2).

The DENN domain family consists of a group of proteins that share common structural features and have been reported to act as nucleotide exchange factors for small GTPases (Yoshimura et al., 2010). A full DENN module is thought to be composed of three subdomains: the upstream longin/u-DENN, the DENN core, and a downstream region called d-DENN (Levivier et al., 2001). The longin domain typically occurs independently of the others and is responsible for the interaction with small GTPases (Kinch and Grishin, 2006; Schlenker et al., 2006) while the DENN and d-DENN domain usually occur together (Levivier et al., 2001). The core DENN domain is an α/β three-layered sandwich domain with a central sheet of five β strands, whereas the d-DENN subregion has an exclusively α-helical secondary structure. The structural similarity of FLCN and the FNIPs to the DENN domain family suggests that this complex might be closely related with members of the Ras-superfamily of small GTPases. Accordingly, pull down and coimmunoprecipitation assays, and live-cell imaging colocalization analyses demonstrated that FLCN and both FNIP1 and 2 interact with the Rag and the Rab GTPases in mammalian cells (Nookala et al., 2012; Petit et al., 2013; Tsun et al., 2013; Starling et al., 2016; Zhao et al., 2018) and in yeast (Péli-Gulli et al., 2017), where they regulate mTORC1 signaling and lysosome positioning respectively.

Two recent cryo-EM structures of the human FLCN-FNIP2-RagA-RagC-Ragulator complex have been determined at a resolution of 3.6 Å (Lawrence et al., 2019) and 3.3 Å (Shen et al., 2019), respectively. Although both density maps are missing important flexible regions, they support a previously unreported atomic model for the full FLCN-FNIP complex. These structures confirm previous computational studies and agree with previous crystal structures (FLCN DENN domain PDB:3V42, RMSD: 1.025 Å, and Lst4 longin domain PDB: 4ZY8, RMSD: 1.869 Å), implying that both FLCN and FNIP contain a DENN module formed by a N-terminal longin domain and a C-terminal DENN domain (Figures 4A,B). Based on homology models and previous structures of certain domains, both studies managed to allocate the structural elements to the density map. The full complex shows an elongated architecture with the Rag GTPases on the middle, contacting Ragulator with their CTDs, as reported by previous structures (de Araujo et al., 2017; Yonehara et al., 2017) (Figure 4B). Both structures show that FLCN and FNIP heterodimerize through their N-terminal longin domains (Figures 4A,B), displaying a comparable architecture to that found in the two longin domains of GATOR1 (Shen et al., 2018). Both longin domains contact the NBDs of both Rag GTPases, establishing more extensive contacts with RagA, whereas the DENN domains interact between them on the opposite site of the structure, far away from the Rag GTPases and Ragulator. The FLCN DENN and longin domains are physically separated with a flexible domain linker in the middle, while the domains of FNIP establish molecular interactions between them (Shen et al., 2019).

Although FLCN is a GAP for RagC, in both structures it is contacting both Rag GTPases, suggesting a unique molecular mechanism for the catalysis of the reaction. The FLCN longin domain directly contacts the NBD of RagA, while FNIP2 longin interacts with the NBD of RagC. Both of them contact their respective Rag GTPase through a complex network of interactions that stably dock the heterodimeric longin domain between the Rag GTPase NBDs (Shen et al., 2019). The architecture of the full complex also explains how the nucleotide state of the Rag GTPases controls recruitment of FLCN to the lysosome: under nutrient rich conditions, the FLCN-FNIP complex is unable to bind to the active Ragulator-Rag complex, thus explaining its cytosolic localization, whereas, upon starvation, the inactive Rag GTPase heterodimer is reoriented, enlarging the cleft formed between the NBDs and leaving enough space for the binding of the FLCN-FNIP complex (Supplementary Figures 3C,D). These novel structures also reveal that FLCN contacts the Rag GTPases heterodimer through their NBDs, using the same interface as mTORC1, therefore providing the explanation for the dissociation of FLCN from the lysosome upon amino acid sufficiency.

A structural comparison between the GATOR1 and FLCN-FNIP complexes, combined with GTPase assays, led to the identification of FLCN-Arg164 as the catalytic residue exerting the GAP activity toward RagC, as mutations affecting this residue reduced the nucleotide exchange rate 100-fold (Lawrence et al., 2019; Shen et al., 2019). However, this residue is pointing away from the RagC nucleotide binding site, thus explaining the lack of GAP activity when FLCN is recruited to the lysosome under nutrient-starved conditions (Figure 4B, *inset*). Despite both structures having reported the same organization of the principal proteins integrating this nonameric complex and the same catalytic residue for FLCN, it is noticeable that the structure of *Lawrence* and colleagues is 20 Å longer than that presented by *Shen* and colleagues. These differences within the same complex indicate that it is quite flexible and oscillates between different transition states, possibly in order to finely regulate the nucleotide state of the Rag GTPases.

## Concluding Remarks

Organelle dynamics in response to nutrient signaling seem to have a great impact on a wide range of cellular functions. FLCN protein, the product of the BHD causative gene, seems to represent one of the most important links between the regulation of lysosome positioning and the regulation of growth through nutrient signaling due to its ability to interact with both Rab and Rag GTPases in response to nutrient availability. The recent structure of the FLCN-Rag-Ragulator complex has shed light on the precise mechanisms by which FLCN regulates Rag GTPase nucleotide state to recruit mTORC1 to the lysosome under conditions of amino acid sufficiency, but there are still many questions that remain unanswered. Several mutations involved in BHD syndrome affect the C-terminal DENN domain of FLCN and, based on the novel structure, these seem very unlikely to disrupt the Rag GTPase interaction with the FLCN complex, indicating that this domain must have other important, as yet undiscovered, functions. Several studies have proposed that the C-terminal DENN domain may be involved in the regulation of membrane trafficking through its interactions with the Rab GTPases, however, definitive support for this notion remains to be found. Intriguingly, FLCN-FNIP can also form complexes with AMPK, reinforcing the importance of the FLCN-FNIP complex in regulating nutrient signaling, however no detailed mechanism is as yet available that might explain the functional relevance of this interaction. Overall, the principle unresolved question that dominates the field is that of how this versatile protein manages to coordinate such a wide range of cellular functions. Structure–function analyses of the FLCN complexes involved in lysosome positioning and AMPK-mTORC1 signaling will continue to provide explanations for the often seemingly disparate phenotypes caused by FLCN loss of function and hopefully contribute to a better insight into the mechanisms involved in the development of BHD-associated and many other tumors.

## Author Contributions

NM wrote the manuscript. CA conceived of the subject matter and reviewed and edited the manuscript.

## Conflict of Interest

The authors declare that the research was conducted in the absence of any commercial or financial relationships that could be construed as a potential conflict of interest.
