# Supplementary material for: Nutrient Signaling and Lysosome Positioning Crosstalk Through a Multifunctional Protein, Folliculin
Source: Front Cell Dev Biol. 2020 Mar 3;8:108. doi: 10.3389/fcell.2020.00108 (PMC7063858; doi:10.3389/fcell.2020.00108)
Supplement: Supplementary file 1 [file Data_Sheet_1.pdf]

Supplementary Material

Nutrient Signaling and Lysosome Positioning Crosstalk Through a Multifunctional Protein, Folliculin

```
FLCN_HUMAN ss:                         β1             β2
FLCN_HUMAN_Q8NFG4             1  M----NAIVALCHFCELHGPRTLFCTEVLHAPLPQGDGNEDSPGQGEQAEEEEGGIQMNSRMRAHSPAEG  66
FLCN_MOUSE_Q8QZS3             1  M----NAIVALCHFCELHGPRTLFCTEVLHAPLPQGAGSGDSPDQVEQAEEEEGGIQMSSRVRAHSPAEG  66
FLCN_Drosophila_Q9VS33        1  M----NAVIALCHFCEAHGPCAIFCTQTLRDTKL------------------------------------  30
FLCN_S.cerevisiae_P53237      1  MS---STVISLAHFCDKHGPRIISVTQSAEKGTL------------------------------------  31
LST7_K.lactis_QEU59399.1      1  MSVDDVGFLSLTHFCDKHGPKVLMVTQMASDLAR------------------------------------  34
Consensus_ss:                          eeeeehhhhhh    eeeeeee

FLCN_HUMAN ss:                                                                    α1
FLCN_HUMAN_Q8NFG4            67  ASVESSSPGPKKSDMCEGCRSLAAGHP--GYISHDKETSIKYVSHQHPSHPQLFSIVRQACVRSLSCEVC  134
FLCN_MOUSE_Q8QZS3            67  ASSESSSPGPKKSDMCEGCRSLAVGHP--GYISHDKETSIKYVSHQHPNHPQLFSIVRQACVRSLSCEVC  134
FLCN_D.melanogaster_Q9VS33   31  ---EDLSLEQQTQKTCSACNYSMGKNN-NAIYSRDTESGATFVSTKVAVLPEVASLVKQAAVLSLSNGTD  96
Lst7_S.cerevisiae_P53237     32  -GEELLVPDYPTESYCESCLLQFPEES--TRSMRCFIEDVPFITTQYS--SIRYQLLNSIIKRAFSEETM  96
Lst7_K.lactis_QEU59399.1     35  -CNELLLPEYPKDSYCESCLLRLPEPKSNATSIRSSIDDFYSVSTQYS---SIRYQLLSMIIKKTFSEET  100
Consensus_ss:                                    hhhh           ee      eeeee   h  hhhhhhhhhhhhhhhh

                                                             Longin
FLCN_HUMAN ss:                         β3        β4                   β5        α2       α3
FLCN_HUMAN_Q8NFG4           135  -PGREGPIFFGDEQHGFVFSHTFFIKDSLARGFQRWYSIITIMMDR--IYLINSWPFLLGKVRGIIDELQ  201
FLCN_MOUSE_Q8QZS3           135  -PGREGPIFFGDEQHGFVFSHTFFIKDSLARGFQRWYSIIAIMMDR--IYLINSWPFLLGRIRGIISELQ  201
FLCN_D.melanogaster_Q9VS33   97  ASKDGEFVFFGDSSRGHILSHTFRVSDLQARGYSQLFSIIVLMKDK--YFLLNIKPFLAEHLKKVSSELQ  164
Lst7_S.cerevisiae_P53237     97  -IYDNMPFIFFDDLRGLNLVIGFKLYDENARGNERRYCFILTVDSR--SHD-DSMKMLSEHWNFIIGGFD  162
Lst7_K.lactis_QEU59399.1    101  MSYDGSPFMFCDEHRGLNLAVGFKLEDVHARGNERRYCLILSLEKRNPCDGNDVFKTLSGNWQFIIESLS  170
Consensus_ss:                          eeeeee    eeeeeeeeee         eeeeeeee  h  hhhhhhhhhhhhhhhhhhhhhh

FLCN_HUMAN ss:                                                                           α4
FLCN_HUMAN_Q8NFG4           202  GKALKVFEAEQFGCPQ--------RAQRM-NTAFTPFLHQRNGNAARSLTSLTSDDNLWACLHTSFAWLL  262
FLCN_MOUSE_Q8QZS3           202  AKAFKVFEAEQFGCPQ--------RAQRM-NTAFTPFLHQRNGNAARSLTSLTSDDNLWACLHTSFAWLL  262
FLCN_Drosophila_Q9VS33      165  AAAKKTKETEEQTYSE--------RQ----RRLSGAQFLM---PTSRALLELTGEEHIFAQLHSHFSWLL  219
Lst7_S.cerevisiae_P53237    163  KMIAYIKNIHKSEFLGKNKTVENNLETLNNNAFIGSYLRANKSKFGRNLVSLTDDKFLFVRIHKWNSFLL  232
Lst7_K.lactis_QEU59399.1    171  KLIDHIKIQAREQLNR--------RQTDFAQIMGGTYLRENKQKLPVSLADIVNDQLIFLRIHKWNTFIL  232
Consensus_ss:                    hhhhhhhhhhhhhh h        hhhh                    eeehe   hhhhhhhhhhhhhh

FLCN_HUMAN ss:
FLCN_HUMAN_Q8NFG4           263  KACGSRLTEKLLEGAPTEDTLVQMEKLADLEEESESWDNSEAEEEEKAPVLPESTEGRELTQ-GPAESSS  331
FLCN_MOUSE_Q8QZS3           263  KACGSRLTEKLLEGAPTEDTLVQMEKLADLEEESESWDNSEAEEEEKAPVTPEGAEGRELTS-CPTESSF  331
FLCN_Drosophila_Q9VS33      220  LAGSRFLTEHVTFGNLPW--------------------------------LPPQSSGRPPAQRLTYNSST  257
Lst7_S.cerevisiae_P53237    233  HTV-------MNENKL------------------------------------------------------  241
LST7_K.lactis_QEU59399.1    233  TRLKSAQAETLDSDGPKD--------------------------------------------------AV  252
Consensus_ss:                    hhh hhhhhhhh

FLCN_HUMAN ss:                              α1             α2          β1        α3         α4
FLCN_HUMAN_Q8NFG4           332  LSGCGSWQPRKLPVFKSLRHMRQVLGAPSFRMLAWHVLMGNQVIWKSRDVDLVQSAFEVLRTMLPVGCVR  401
FLCN_MOUSE_Q8QZS3           332  LSACGSWQPPKLTGFKSLRHMRQVLGAPSFRMLAWHVLMGNQVIWKSRDVNLVHSAFEVLRTMLPVGCVR  401
FLCN_Drosophila_Q9VS33      258  LPMIESIDDPDLEEFFSLRHLKSVVRKEEFATVCYCALTGVKIVVRGD-PRKTFRFMVCLKKLLPEPMHN  326
Lst7_S.cerevisiae_P53237    242  -P--------------------------------------------------------------------  242
Lst7_K.lactis_QEU59399.1    253  KP--------------------------------------------------------------------  254
Consensus_ss:                               hhhhhhhhhhhhhh hhhhhhhhhhhhh  eeeeee  hhhhhhhhhhhhhh  hhhh

                                                              DENN
FLCN_human ss:                        α5                 α6   β2                           β3
FLCN_HUMAN_Q8NFG4           402  IIPYSSQYEEAYRCNFLGLSPHVQIPPHVLSSEFAVIVEVHAAARSTLHPVGCEDDQSLSKYEFVVTSGS  471
FLCN_MOUSE_Q8QZS3           402  IIPYSSQYEEAYRCNFLGLSPPVPIPAHVLASEFVVVVEVHTATRSNLHPAGCEDDQSLSKYEFVVTSGS  471
FLCN_Drosophila_Q9VS33      327  LMRIDAQHQHSISSEYKIISVSNDIAVPMASSSVYRIDFLD---------------KHVNGHE--VSVKW  379
Lst7_S.cerevisiae_P53237         ----------------------------------------------------------------------
Lst7_K.lactis_QEU59399.1         ----------------------------------------------------------------------
Consensus_ss:                         hh   hhh                    eee                             eee

FLCN_HUMAN ss:                              α7                      α8                       α9
FLCN_HUMAN_Q8NFG4           472  PVAADRVGPTILNKIEAALTNQNLSVDVVDQCLVCLKEEWMNKVKVLFKFTKVDSRPKEDTQKLLSILGA  541
FLCN_MOUSE_Q8QZS3           472  PVAADRVGPTILNKIEAALTNQNLSVDVVDQCLICLKEEWMNKVKVLFKFTKVDSRPKEDTQKLLSVLGA  541
FLCN_Drosophila_Q9VS33      380  PGELPRKLPDLMVKLLKAVEEKNFTELVLNKQTKVLIEEWKNKVTCLNHAKST-----SVQGKLKKVLGV  444
Lst7_S.cerevisiae_P53237         ----------------------------------------------------------------------
Lst7_K.lactis_QEU59399.1         ----------------------------------------------------------------------
Consensus_ss:                             hhhhhhhhhhhh    hhhhhhhhhhhhhh    eeeeeee           hhhhhhh
```

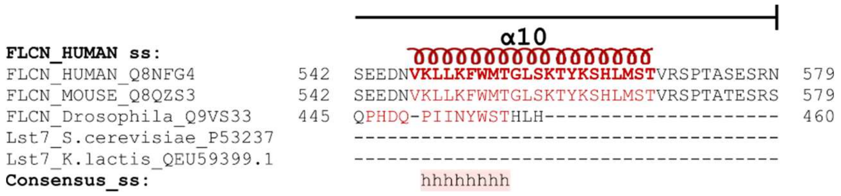

Supplementary Figure 1: Alignment of multiple FLCN sequences with their respective yeast orthologue, Lst7. Multiple sequence alignment and secondary structure prediction of FLCN sequences from different species were made with PROMALS3D software (Pei, Kim, and Grishin 2008). Blue residues correspond to putative β-strands while red residues to possible α-helices. The consensus secondary structure is represented following the next code: e corresponds to β-strands and h to α-helices. Basic structural elements and domains based on the two recent human FLCN-FNIP2 structures (PDB: 6NZD, PDB: 6ULG) (Lawrence et al. 2019; Shen et al. 2019) are displayed on the top of the alignment. Protein sequences available in Uniprot (FLCN human: Q8NFG4, FLCN mouse: Q8QZS3, FLCN *D.melanogaster* Q9VS33, Lst7 S.cerevisiae P53237) and GenBank (Lst7 *K.lactis* QEU59399.1).

```
FNIP2_HUMAN ss:
FNIP2_HUMAN_9P278                  ----------------------------------------------------------------------
FNIP2_MOUSE_Q80TD3                 ----------------------------------------------------------------------
GH23617p_D.melanogaster_Q          ----------------------------------------------------------------------
Lst4_S.cerevisiae_P34239_     1    MLGNLLRNKTSSSGFEKSSEHSDFSSVVPNVPVYCKAASTGTTKTAAGALLDTAVNVEKPSEMLSTTSPP    70
Lst4_K.lactis_Q6CXP4          1    MLGRLLRT-------------SSLSEFVPF----------GSS------------NVTVGEEVFQNEP--    33
Consensus_ss:                        hhhh                    hhhh

                                                                                β1
FNIP2_HUMAN ss:
FNIP2_HUMAN_9P278             1    ----MAPTLLQKLFNKRGSSGSSAAASAQGRAPKEGP---AFSWSCSE----FDLNEIRLIVYQDC----    55
FNIP2_MOUSE_Q80TD3            1    ----MAPTLLQKLFNKRGGG----AASAQARPPKEEP---AFSWSCSE----FGLSDIRLLVYQDC----    51
GH23617p_D.melanogaster_Q     1    ----M-----------------------------------HTPPFKSNRFPFEGSQVRVLLYKE-----    25
Lst4_S.cerevisiae_P34239_    71    ILDHISDDLKLKLFGSRDI-----PYSRPIDTLQNNGGLGTDKITSIN----EKTYAFRILIIEEAGQMA   131
Lst4_K.lactis_Q6CXP4         34    --FYMTDDLKTLLYGTRDKA----LFERIANDRLH------------------NGGFRLIISQELGHVT    78
Consensus_ss:                            hhhhhheee                                        eeeeeeeehhhhhh

                                        β2
FNIP2_HUMAN ss:
FNIP2_HUMAN_9P278            56    DRRGRQVLFDSKAVQKIEEVTAQKTEDVPIK--------ISAKCCQGSSSVSSSSSSSISSHSSSGGSSH   117
FNIP2_MOUSE_Q80TD3           52    ERRGRQVMFDSRAVQKMEEAAAQKAEDVPIK--------MSARCCQESSSSSGSSSSGSSSSHGFGGSLQ   113
GH23617p_D.melanogaster_Q    26    -DDTRRLLFDSNALQKVMHKD-QSATSASISSTSTSGGGKFLKNEKYTSLQNGKIPSKSHSSN---GSNF    90
Lst4_S.cerevisiae_P34239_   132    CRNNYRDIFDYTTSK--------------ISNSM-----------------------------------   151
Lst4_K.lactis_Q6CXP4         79    SRNNYQVVLDHSSVN---------------FHTG-----------------------------------    97
Consensus_ss:                      hh    eeeeee  hh

                                                        Longin
                                                  α1               β3         β4
FNIP2_HUMAN ss:
FNIP2_HUMAN_9P278           118    HAKEQLPKYQYTRPA-SDVNMLGEMMFGS-VAM-SYKGSTLKIHYIRSPPQLMISKVFSARMGS------   178
FNIP2_MOUSE_Q80TD3          114    HAKQQLPKYQYTRPA-SDVSMLGEMMFGS-VAM-SYKGSTLKIHYIRSPPQLMISKVFSATMGS------   174
GH23617p_D.melanogaster_Q    91    IEVCAEYGYKHNRPSGADITPVGEMVFGS-LPM-SFCGTALKVHWLPEPSRILCSQVYLTPTSNGGSGHS   158
Lst4_S.cerevisiae_P34239_   152    --------------EQIRPSELKEYIFGSPVRSSD-LTQCDKIRTIPNSDLVLITRIFYYTHQ-------   199
Lst4_K.lactis_Q6CXP4         98    --------------AHIPLNELKDYIFGSSIRTIDYSASSDKIKVVKSANIVLFTRIFYLNEK-------   146
Consensus_ss:                                     hhhhhhhhh                eeeeee     eeeeeeeeee

Disordered region

                                                          β5             α2          α3
FNIP2_HUMAN ss:
FNIP2_HUMAN_9P278           287    STDETFSLAEETCSSNPA---MVRRKKIAISIIFSLCEKEEAQRNFQDFFFSHFPLFESHMNRLKSAIEK   353
FNIP2_MOUSE_Q80TD3          283    STDETFSLAEETCSSNPA---MVRRKKIAISIIFSLCEREAAQRDFQDFFFSHFPLFESHMNRLKGAIEK   349
GH23617p_D.melanogaster_Q   362    YRRASYCANETRSNPEMGRRQANLRRRAKLGLAVCISMSESFEEEMELFCSEHIALLESMLSRLRASTEL   431
Lst4_S.cerevisiae_P34239_   200    -------------------------YNRIAISLCIP------RILLPVVAESWSSISSWLTQTQKMLIG   237
Lst4_K.lactis_Q6CXP4        147    -----------------------STL-RIAISCCVT------DDVLPVLTECWPHISSFLDQCENTLLK   185
Consensus_ss:                       hh                        eeeeeeee    hhhhhhhhhhhhhhhhhhhhhhhhhhhhhh

                                                              α4
FNIP2_HUMAN ss:
FNIP2_HUMAN_9P278           354    AMISCRK-------------IAESSLRVQ------FYVSRLMEALGEFR----GTIWNLYSVPRIAEPVW   400
FNIP2_MOUSE_Q80TD3          350    AMISCRK-------------ISESSLRVQ------FYVSRLMEALGEFR----GTIWNLYSVPRIAEPVW   396
GH23617p_D.melanogaster_Q   432    AYINHKN------------------------------FLQIMFQAWQDTQ----QWFSDLFTAPRIKTPVW   468
Lst4_S.cerevisiae_P34239_   238    FLTKNRIMQENTGNYSNNSVIKL---SNIDIRTHYP--KEIEIMVQTLQKRVIPGLRSMSEIPRL-----   297
Lst4_K.lactis_Q6CXP4        186    YLAKND--------------TQF---LPHDWKARNC--IEVAAVLQTFQRKIIPLLSGYSDTPRL-----   231
Consensus_ss:                      hhhhh                     h        hhhhhhhhhhhhhhhhhhhhhhhhh          ee

                                              α1                       α2
FNIP2_HUMAN ss:
FNIP2_HUMAN_9P278           401    LTMMSG-TLEKNQLCQRFLKEFTLLIEQIN---KNQFFAALLTAVLTYHLAWVPTVMPVDHPPIKAFSEK   466
FNIP2_MOUSE_Q80TD3          397    LTMMSN-TLEKNQLCQRFLKEFILLIEQVN---KNQFFAALLTAVLTYHLAWVPTVMPVDHPPIKAFSEK   462
GH23617p_D.melanogaster_Q   469    LSITTSGSKYSKTVAERFIKELCDLLSFADTKDSNFFISTMLTGILTHHLGWVATVSAFNSSGSKRSESS   538
Lst4_S.cerevisiae_P34239_   298    ---FLY-PETFKEFVHVWFKSIFNWIEIKDG-PKLGFLPLLMAMIISDYRHTIREL--------------   348
Lst4_K.lactis_Q6CXP4        232    ---FLY-PMDSIPYIKTWVKYVTNWIELKDG-PRVRFLPILLAKLRYDFASLLKEN--------------   282
Consensus_ss:                                  hhhhhhhhhhhhhhhhhh      hhhhhhhhhhhhhhhhhhhh

                                                                              β1          α3
FNIP2_HUMAN ss:
FNIP2_HUMAN_9P278           467    RTSQSVNMLA------KTHPYNPLWAQLGDLYGAIGSPVRLTRTVVVGKQKDLVQRILYVLTYFLRCSEL   530
FNIP2_MOUSE_Q80TD3          463    RTSQSVNMLA------KTHPYNPLWAQLGDLYGAIGSPVRLTRTVVIGKQKDLVQRILYVLTYFLRCSEL   526
GH23617p_D.melanogaster_Q   539    ASAAAIEQRAKLLQVAQKHPYNALWAQLGDLYGAIGMPPKLARTIVCGAEKLWVEKLLNVLTYFIRCSEV   608
Lst4_S.cerevisiae_P34239_   349    ---------------------------------------KTSKIVILSGNMVVANKLLFILSALLEPK--   377
Lst4_K.lactis_Q6CXP4        283    ---------------------------------------SNTRIVILTGNMNVANRLIFILTAFLGPH--   311
Consensus_ss:                       hhhhhhhh                    hhhhhhhhhh      eeeeeeee   hhhhhhhhhhhhhhhhhhhhh
```

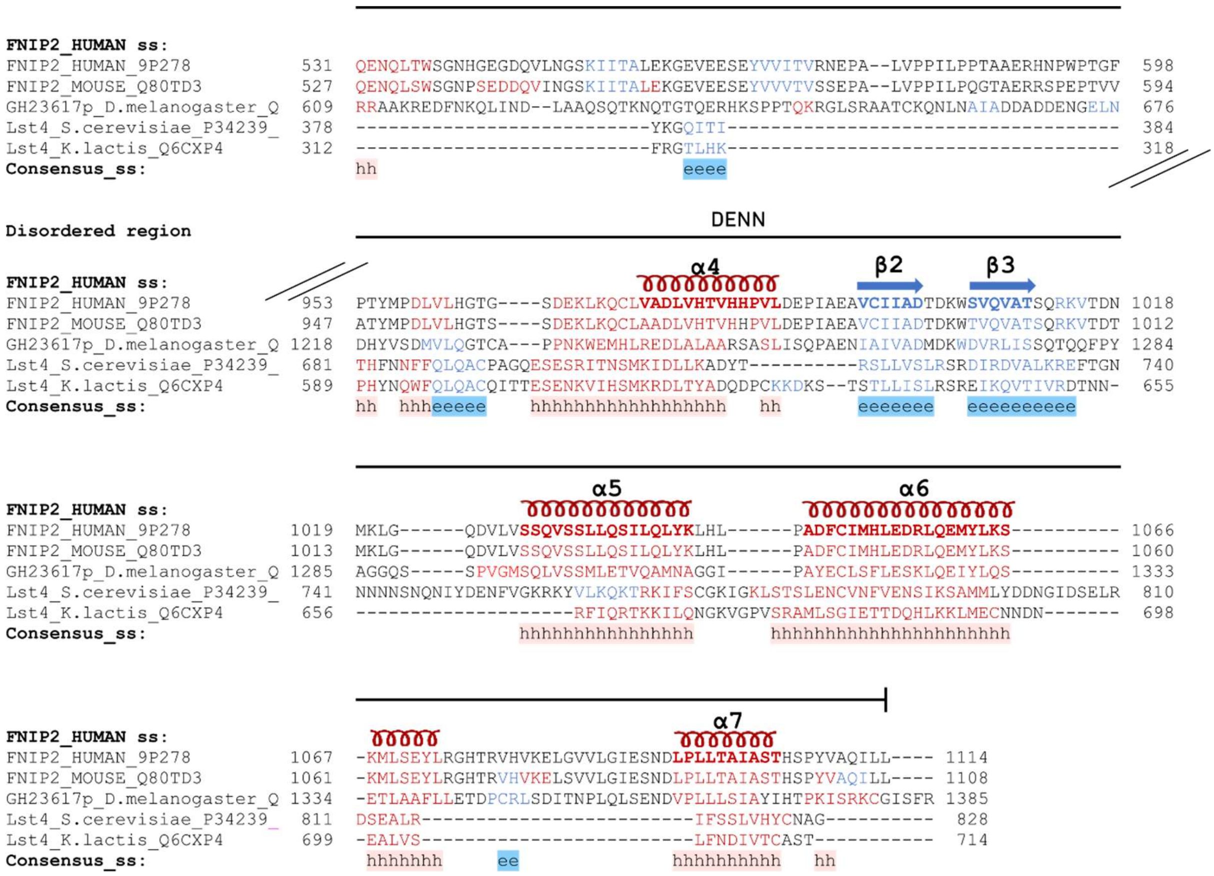

Supplementary Figure 2: Alignment of multiple FNIP2 sequences with their respective yeast orthologue, Lst4. Multiple sequence alignment and secondary structure prediction of FNIP2 sequences from different species were made with PROMALS3D software (Pei, Kim, and Grishin 2008). Blue residues correspond to putative β-strands while red residues to potential α-helices. The consensus secondary structure is represented following the next code: e corresponds to β-strands and h to α-helices. Basic structural elements and domains based on the two recent human FLCN-FNIP2 structures (PDB: 6NZD, PDB: 6ULG) (Lawrence et al. 2019; Shen et al. 2019)are displayed on the top of the alignment. Protein sequences from Uniprot (FNIP2 human: Q9P278, FNIP2 mouse: Q80TD3, GH23617p *D.melanogaster* Q8T0L7, Lst4 S.cerevisiae P34239, Lst7 *K.lactis* Q6CXP4).

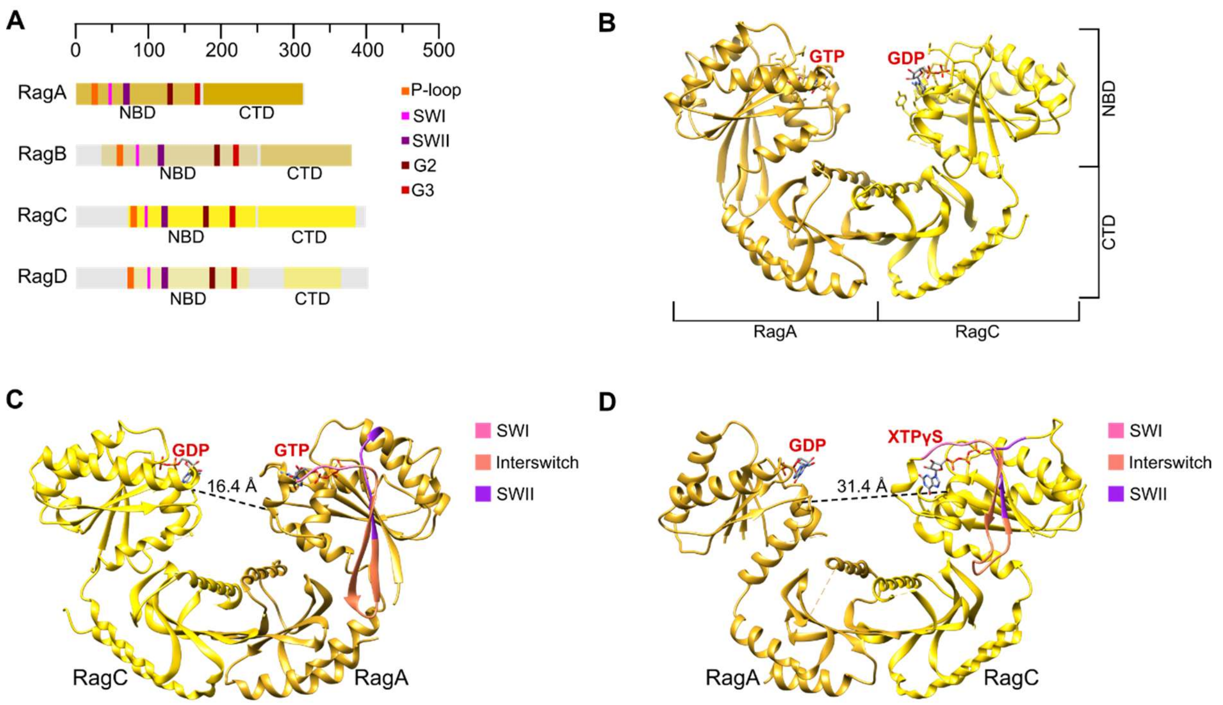

Supplementary Figure 3: The Rag GTPases. (**A**) Schematic representation of the human Rag GTPases with domains coloured accordingly and typical regulatory elements from GTPases highlighted in small boxes. Top ruler represents number of amino acids. (**B**) RagA-RagC heterodimer structure with GTP and GDP bound respectively (PDB: 6U62) (Rogala et al. 2019). Both Rag GTPases are shown in cartoon representation, with RagA shown in gold and RagC in yellow. (**C**) Structure of the RagA-RagC heterodimer on its active nucleotide state (PDB: 6U62) (Rogala et al. 2019). Switch and interswitch regions from RagA-GTP are coloured according to the legend on the left. (**D**) Structure of the RagA-RagC heterodimer when it forms complex with Ragulator and FLCN-FNIP2 (PDB: 6NZD) (Lawrence et al. 2019). In this case, the Rag GTPase heterodimer is on its inactive nucleotide binding state, that is RagA-GDP and RagC-GTP (XTPγS). Switch and interswitch regions from RagC-GTP are coloured according to the legend on the right.

References

Lawrence, Rosalie E., Simon A. Fromm, Yangxue Fu, Adam L. Yokom, Do Jin Kim, Ashley M. Thelen, Lindsey N. Young, et al. 2019. "Structural Mechanism of a Rag GTPase Activation Checkpoint by the Lysosomal Folliculin Complex." *Science*, October, eaax0364. https://doi.org/10.1126/science.aax0364.

Pei, Jimin, Bong-Hyun Kim, and Nick V. Grishin. 2008. "PROMALS3D: A Tool for Multiple Protein Sequence and Structure Alignments." *Nucleic Acids Research* 36 (7): 2295–2300. https://doi.org/10.1093/nar/gkn072.

Rogala, Kacper B., Xin Gu, Jibril F. Kedir, Monther Abu-Remaileh, Laura F. Bianchi, Alexia M. S. Bottino, Rikke Dueholm, et al. 2019. "Structural Basis for the Docking of MTORC1 on the Lysosomal Surface." *Science*, October, eaay0166. https://doi.org/10.1126/science.aay0166.

Shen, Kuang, Kacper B. Rogala, Hui-Ting Chou, Rick K. Huang, Zhiheng Yu, and David M. Sabatini. 2019. "Cryo-EM Structure of the Human FLCN-FNIP2-Rag-Ragulator Complex." *Cell* 179 (November): 1–11. https://doi.org/10.1016/j.cell.2019.10.036.
